# COP9 signalosome subunit 6 (CSN6) regulates E6AP/UBE3A in cervical cancer

**DOI:** 10.18632/oncotarget.4731

**Published:** 2015-08-03

**Authors:** Shujun Gao, Lekun Fang, Liem Minh Phan, Aiham Qdaisat, Sai-Ching J. Yeung, Mong-Hong Lee

**Affiliations:** ^1^ Obstetrics and Gynecology Hospital Fudan University, Shanghai Key Laboratory of Female Reproductive Endocrine Related Diseases, Shanghai 200011, China; ^2^ Department of Molecular and Cellular Oncology, The University of Texas MD Anderson Cancer Center, Houston, Texas 77030, USA; ^3^ Department of Colorectal Surgery, Guangdong Provincial Key laboratory of Colorectal and Pelvic Floor Diseases, The Sixth Affiliated Hospital of Sun Yat-sen University, Guangzhou 510655, China; ^4^ Department of Endocrine Neoplasia and Hormonal Disorders, The University of Texas MD Anderson Cancer Center, Houston, Texas 77030, USA; ^5^ Department of Emergency Medicine, The University of Texas MD Anderson Cancer Center, Houston, Texas 77030, USA; ^6^ Program in Cancer Biology, The University of Texas Graduate School of Biomedical Sciences at Houston, Houston, TX 77030, USA; ^7^ Program in Genes and Development, The University of Texas Graduate School of Biomedical Sciences at Houston, Houston, TX 77030, USA

**Keywords:** CSN6, E6AP, p53, cervical cancer, ubiquitination

## Abstract

Cervical cancer is one of the leading causes of cancer death in women. Human papillomaviruses (HPVs) are the major cause in almost 99.7% of cervical cancer. E6 oncoprotein of HPV and E6-associated protein (E6AP) are critical in causing p53 degradation and malignancy. Understanding the E6AP regulation is critical to develop treating strategy for cervical cancer patients. The COP9 signalosome subunit 6 (CSN6) is involved in ubiquitin-mediated protein degradation. We found that both CSN6 and E6AP are overexpressed in cervical cancer. We characterized that CSN6 associated with E6AP and stabilized E6AP expression by reducing E6AP poly-ubiquitination, thereby regulating p53 activity in cell proliferation and apoptosis. Mechanistic studies revealed that CSN6-E6AP axis can be regulated by EGF/Akt signaling. Furthermore, inhibition of CSN6-E6AP axis hinders cervical cancer growth in mice. Taken together, our results indicate that CSN6 is a positive regulator of E6AP and is important for cervical cancer development.

## INTRODUCTION

Cervical cancer is the leading cause of women's cancer death. Most of the cervical cancers are caused by the infection of the human papillomavirus (HPV). HPV E6 and cellular E6-Associating Protein (E6AP/UBE3A) proteins target p53 for proteasome-mediated degradation, thereby facilitating cellular transformation [1, 2]. E6AP has a role in numerous diseases including neurological disorders and cancers. Neuron-specific loss of E6AP leads to Angelman syndrome (AS) [3]. E6AP expression is elevated in 60% of human Burkitt lymphomas [4], and its inhibition is critical in Tamoxifen-mediated antibreast cancer actions [5]. As an E3 ubiquitin ligase, E6AP has many substrates. In addition to p53, it also has a role in regulating Tert [6], beta-catenin level [7], ErBb2 [8], PML [9], peroxiredoxin 1 [10], CCAAT/Enhancer Binding Protein Alpha (C/EBPalpha), Blk [11], and TSC2 [12]. Also, the molecular regulations of E6AP in cancers have been studied, yielding discoveries such as its regulation by HMGB2 [13], c-abl [14], miR-375 [15], and E6 [16]. However, a detailed picture of the pathways regulating E6AP has yet to emerge. Defining these molecular regulations can help guide treatment and improve clinical care of tumors including cervical cancer.

The COP9 signalosome (CSN) is a protein complex involved in protein degradation, transcriptional activation [17, 18], signal transduction [19–22], DNA damage response/genome integrity [23–25], and tumorigenesis [21, 23, 26–29]. The contribution of the CSN's subunits in cancer has not been well characterized. Mammalian CSN subunits are critical in developmental processes. Knockout of mammalian *Csn2, Csn3, Csn5*, and *Csn8* caused defective embryo development [30–33]. We previously studied targeted disruption of the *Csn6* gene in mice and found that *Csn*6–/– mice developed until 7.5 days post-coitus [28]. In a *Csn6*+/– mouse tumor experiment we showed that *Csn6* haplo-insufficiency mitigated the development of cancer [28], which indicates that CSN6 expression level is important for tumorigenesis. Several studies show that CSN6 expression is elevated in cancers and leads to poor survival [21, 34, 35], suggesting that abnormal CSN6 overexpression allows cancer to have growth advantages. However, the level and biological consequence of CSN6 expression in cervical cancer remain unclear.

In the present study, we found that CSN6 is overexpressed in cervical cancer and is an important positive regulator of E6AP. We also characterized that CSN6-E6AP axis is regulated by EGF signaling. Our studies provide important insight into the signaling of the CSN6-E6AP axis in the tumorigenesis of cervical cancer and help elucidate CSN6’s potential as a target for therapeutic design for cervical cancer.

## RESULTS

### CSN6 interacts with E6AP *in vivo* and causes E6AP stabilization

**Figure 1:**
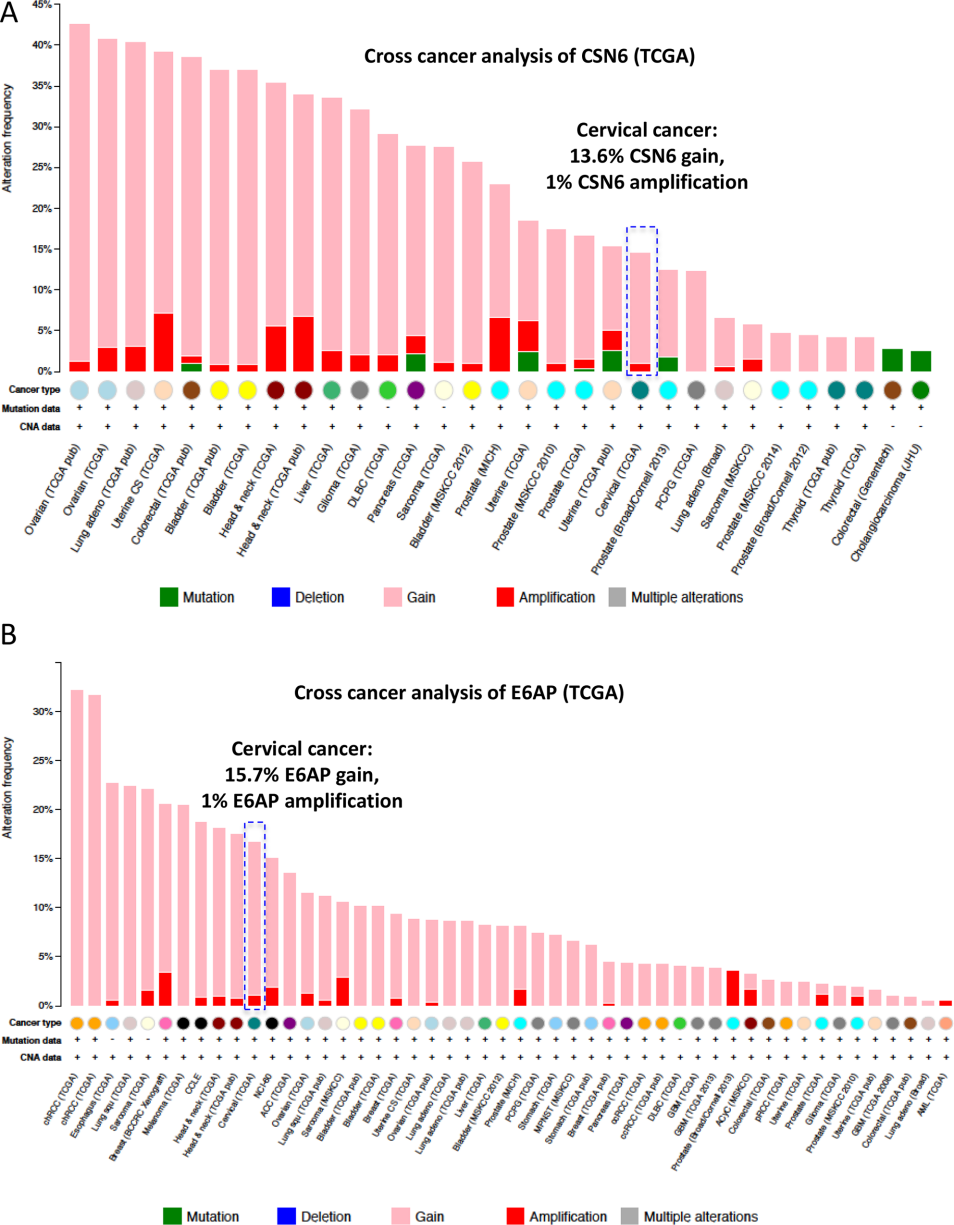
Cross-cancer genomic analysis of CSN6 and E6AP using TCGA **A.** Cross cancer genomics analysis of CSN6 (TCGA database, cBioportal). CSN6 gain and amplification are frequently observed in multiple common types of cancer. **B.** Cross cancer genomics analysis of E6AP (TCGA database, cBioportal). E6AP gain and amplification are common in many types of cancer.

**Figure 2:**
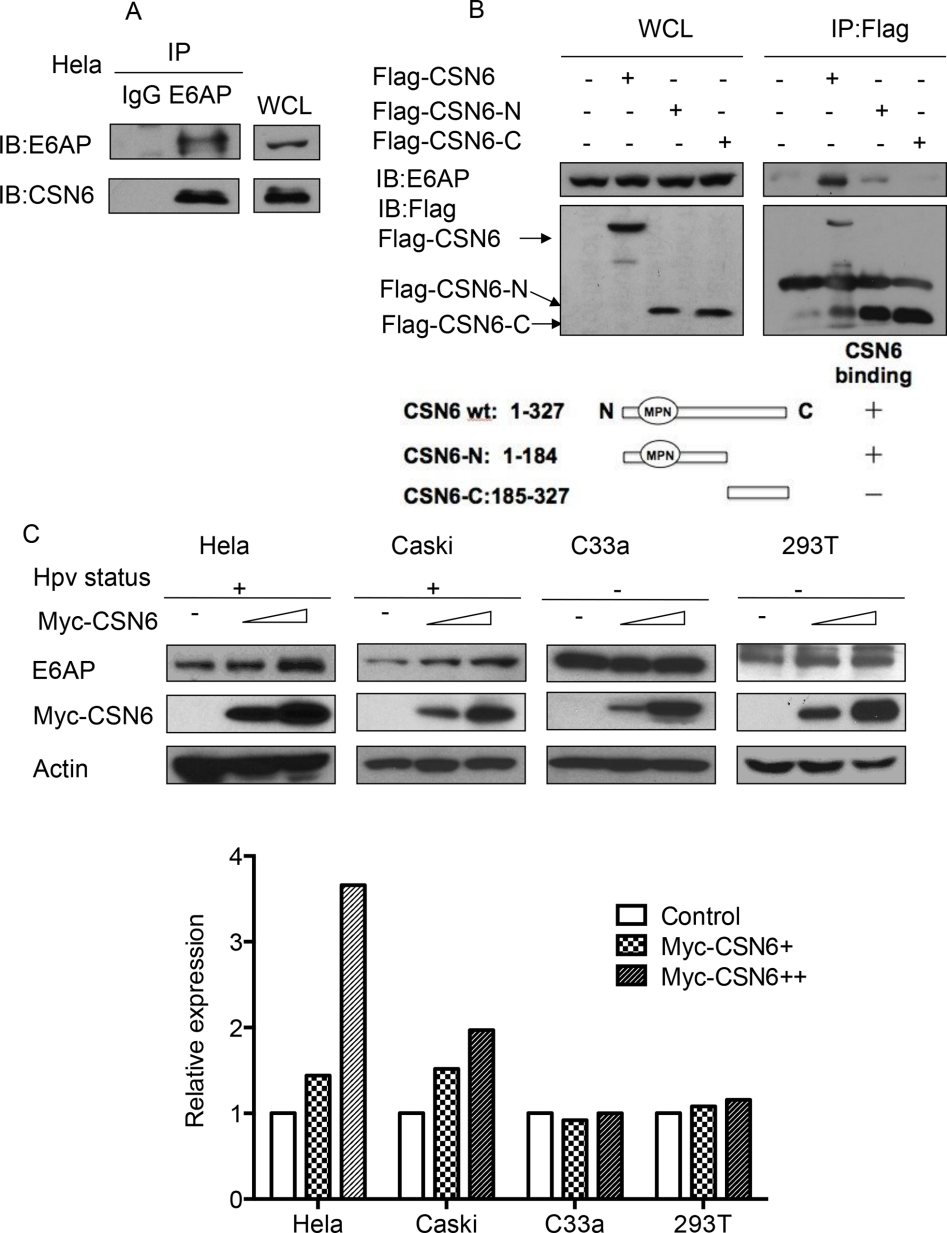
CSN6 interacts with E6AP *in vivo* and causes E6AP stabilization **A.** Physical interaction of endogenous CSN6 with endogenous E6AP. Equal amounts of HeLa cells lysates were immunopricipitated with either mouse IgG or anti-E6AP antibody and immunoblotted with the indicated antibodies. Whole cell lysates (WCL) were immunoblotted with indicated antibody. **B.** Mapping of E6AP binding domain on CSN6. Flag-CSN6 (aa 1–327), N-terminus (aa 1–184), C-terminus (aa 185–327) were transfected into HeLa cells respectively. Cell lysates were immunoprecipitated with anti-Flag and then immunoblotted with anti-E6AP antibody. Whole cell lysates (WCL) were immunoblotted with indicated antibody. **C.** CSN6 increases the steady-state expression of E6AP protein in HPV positive cell lines (HeLa and Caski cells) in a dose-dependent manner. HeLa, Caski, C33A and 293T cells were transfected with a fixed amount of expression vector for Myc-CSN6 (10 μg) and increasing amounts of Myc-CSN6 (10 μg, 20 μg). Total cell lysates were harvested and analyzed by immunoblotting with anti-E6AP antibody. Relative expression of E6AP from each group was measured and presented as a bar graph.

To understand the gene status of CSN6 and E6AP in cancers, we performed a cross-cancer genomic analysis of CSN6 and E6AP using The Cancer Genome Atlas (TCGA) database. We found that both CSN6 and E6AP are overexpressed in many types of cancer, including cervical cancer (Figure 1). On the basis of these findings, we hypothesized that CSN6 and E6AP have an interactive or regulatory relationship. Co-immunoprecipitation experiments showed endogenous interaction of the two proteins in cells (Figure 2A). Next, we mapped the structural regions of CSN6 required for its interaction with E6AP in a co-immunoprecipitation experiment. Results showed that E6AP was bound to the MPN (Mpr1p and Pad1p N-terminal) domain containing N-terminus of CSN6 (aa 1–184 containing MPN motif) but not to the C-terminus (aa 185–327; Figure 2B). We then showed that CSN6 was able to upregulate steady-state expression of E6AP in a dose-dependent manner in HPV positive cells (HeLa and Caski), while its impact in HPV negative cells (C33A and 293T) was not that obvious (Figure 2C). These results indicate that CSN6 associates with E6AP and that E6AP can positively affect the steady-state expression of CE6AP.

### CSN6 increases E6AP stability by reducing E6AP poly-ubiquitination

**Figure 3:**
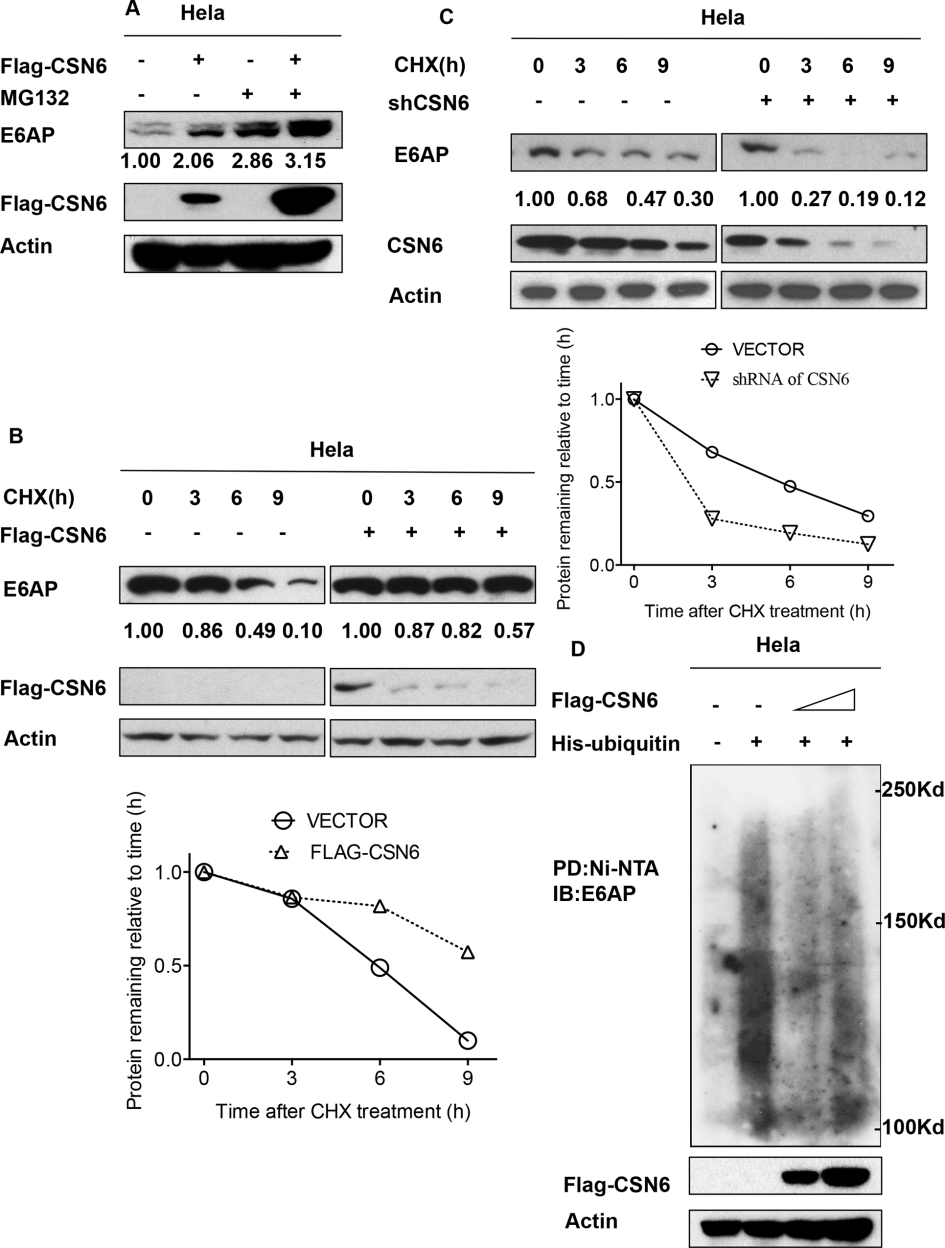
CSN6 increases E6AP stability by reducing E6AP poly-ubiquitination **A.** CSN6-mediated stabilization of E6AP is proteasome-dependent. HeLa cells were transfected with Flag-CSN6 or vector control transfectant. Cells were treated with or without MG132 for 6 h before harvesting. Equal amount of cell lysates were immunoblotted with the indicated antibodies. **B.** and **C.** E6AP turnover rate is changed in CSN6 overexpressing or knock down HeLa cells. HeLa cells were transfected with indicated plasmids and treated with cycloheximide (CHX) (100 μg /ml) for the indicated times. Cell lysates were immunoblotted with the indicated antibodies. Integrated OD values of bands at each time point were measured by imageJ. Levels of E6AP at time zero were set at 100%. Remaining E6AP is indicated graphically. **D.** CSN6 decreases endogenous E6AP poly-ubiquitination. HeLa cells were transfected with increasing CSN6 expressing plasmids. MG132 was added 6 h before they were harvested with guanidine-HCl-containing buffer. The cell lysates was then pulled down with nickel beads and immunoblotted with anti-E6AP antibody. Equal amount of whole cell lysates were immunoblotted with anti-CSN6 or Actin.

We found that CSN6-mediated E6AP upregulation was enhanced by the proteasome inhibitor MG132 (Figure 3A). These results suggest that CSN6 increased steady-state expression of E6AP in a proteasome-dependent manner. CSN6 decreased the turnover rate of E6AP in the presence of the *de novo* protein synthesis inhibitor cycloheximide (Figure 3B), whereas the shRNA of CSN6 increase turnover of E6AP (Figure 3C). Congruently, we then found that overexpression of CSN6 decreased the endogenous ubiquitination level of E6AP (Figure 3D). Together, these results suggest that CSN6 is critical for the stability of E6AP.

### CSN6 knockdown affects E6AP-p53 axis and cell proliferation, cell motility, and apoptosis

**Figure 4:**
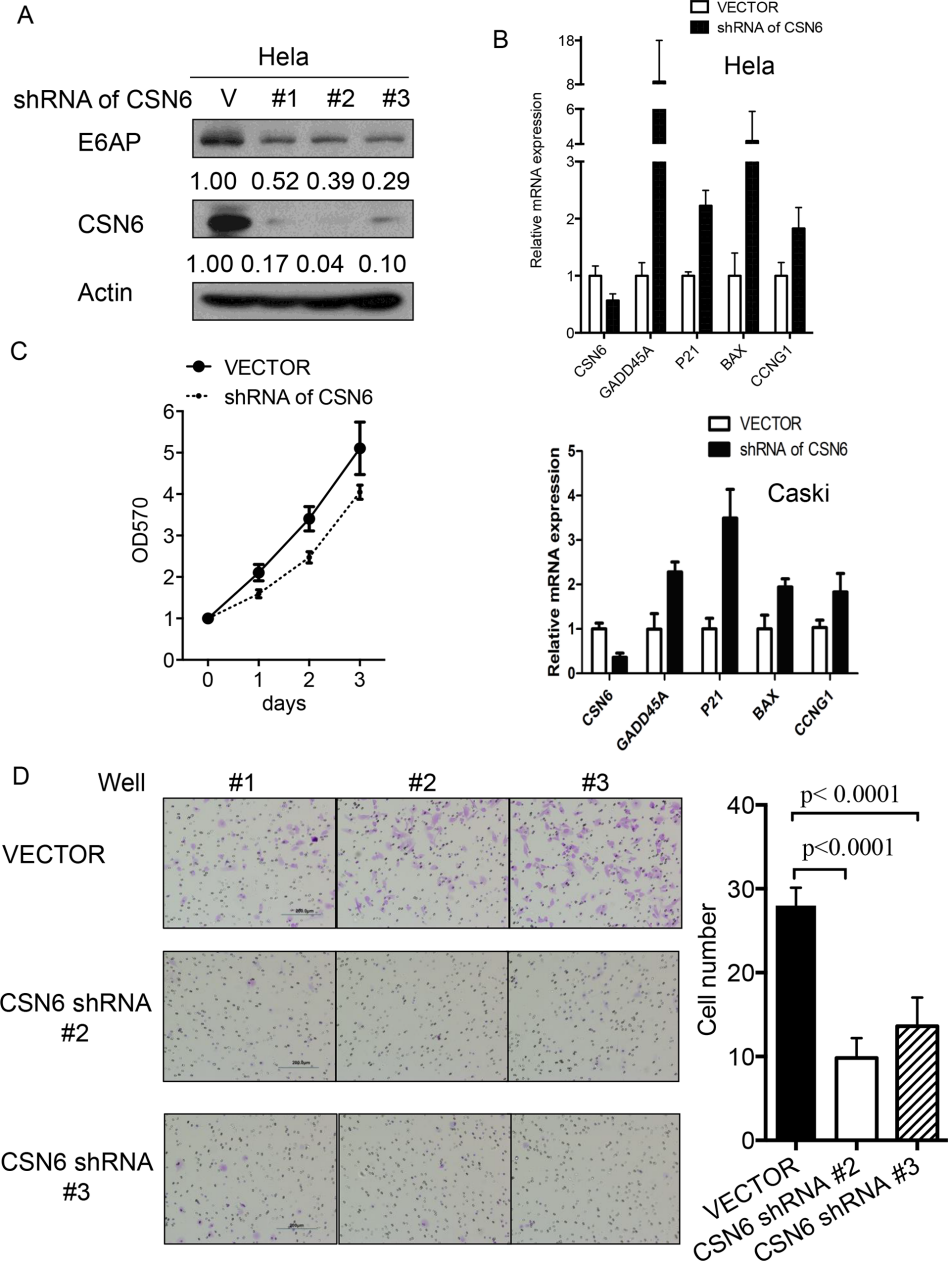
CSN6 knockdown affects E6AP-p53 axis and cell proliferation **A.** E6AP protein is downregulated when endogenous CSN6 expression is inhibited with shRNA. Lysates of HeLa cells infected with either CSN6 shRNA(#1, #2, #3) or control shRNA(V) were immunoblotted with indicated antibodies. **B.** RNA was extracted from HeLa or Caski cells infected with either CSN6 shRNA or control shRNA (vector). qRT-PCR analysis was performed to measure the mRNA level of p53 transcriptional target genes. Data are means ± SDs. **C.** HeLa cells were infected with either CSN6 shRNA or control shRNA (Vector). Cell proliferation rates were measured by MTT assay. The results were measured at an optical density of 570 nm. **D.** Cell motility was analyzed by migration assay. Data represent three independent experiments. Number of migrated cells from each group were measured and presented as a bar graph. Data are means ± SDs.

**Figure 5:**
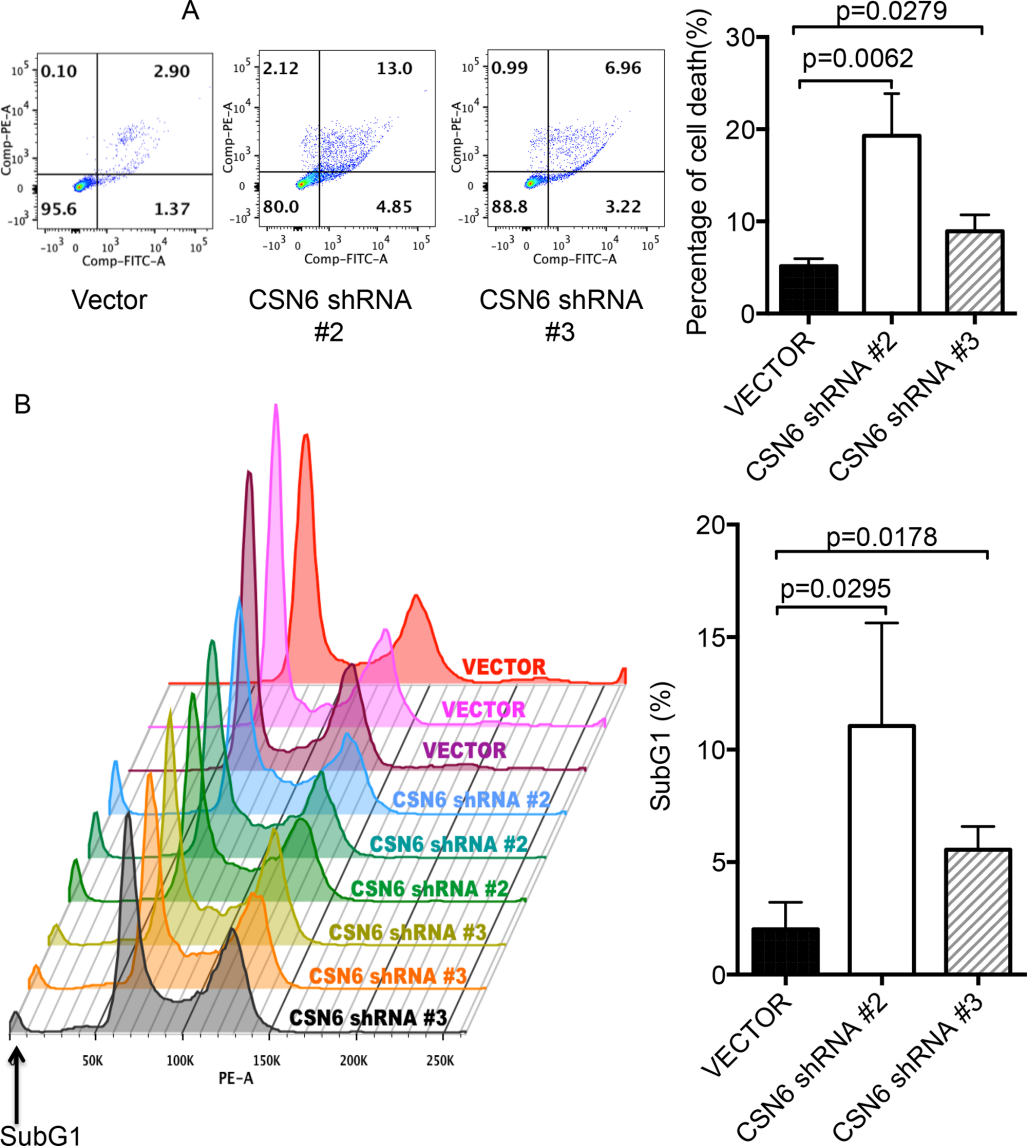
CSN6 knockdown affects E6AP and cell survival **A.** Knockdown of CSN6 accelerated apoptosis. HeLa cells were infected with either CSN6 shRNA or control shRNA (Vector). Binding of Annexin V and uptake of propidium iodide were analyzed by flow cytometry. Left panel is representative analysis of apoptotic cells. The lower left quadrant contains the viable population of cells, the upper left quadrant contains necrotic cells, the lower right quadrant contains early apoptotic cell and the upper right quadrant contains late apoptotic cells. The mean of three data sets was taken and the values shown from the corresponding quadrants (right panel). Error bars represent 95% confidence intervals. **B.** Knockdown of CSN6 increases sub-G1 population in HeLa cells. HeLa cells were infected with either CSN6 shRNA or control shRNA (Vector). After harvesting, cells were fixed and stained with EtOH and propidium iodide (PI), respectively. Stained cells were detected by flow cytometry analysis and analyzed using Flow Cytometry Analysis software. Error bars represent 95% confidence intervals.

E6AP targets p53 for proteasome-mediated degradation, which in turn will reduce the transcriptional activity of p53 target genes involved in cell growth and apoptosis. We examined whether CSN6-E6AP axis could affect cell proliferation. We found that E6AP levels were reduced when cells were treated with CSN6-shRNA virus to perform CSN6 knockdown (Figure 4A). The biological significance of E6AP reduction caused by CSN6 knockdown was an upregulation of p53 target gene expression, including GADD45, p21, and Bax, (Figure 4B), which leads to a slow cell growth as assayed by MTT assay (Figure 4C). CSN6 knockdown also reduced the cell motility of HeLa cells (Figure 4D). Further, we found that CSN6 knockdown affects cell survival. HeLa cells were infected with either CSN6 shRNA or vector control, and binding of Annexin V and uptake of propidium iodide were analyzed by flow cytometry. CSN6 knockdown increased apoptosis as evident in increased Annexin V staining when compared with vector control (Figure 5A). CSN6 knockdown also accelerated apoptosis as evident in increased sub-G1 population after propidium iodide staining followed by flow cytometry (Figure 5B). Together, these data indicate that CSN6-mediated E6AP stabilization is participating in cell growth, cell motility, and apoptosis.

### EGF-Akt signaling regulates CSN6 and E6AP stability

**Figure 6:**
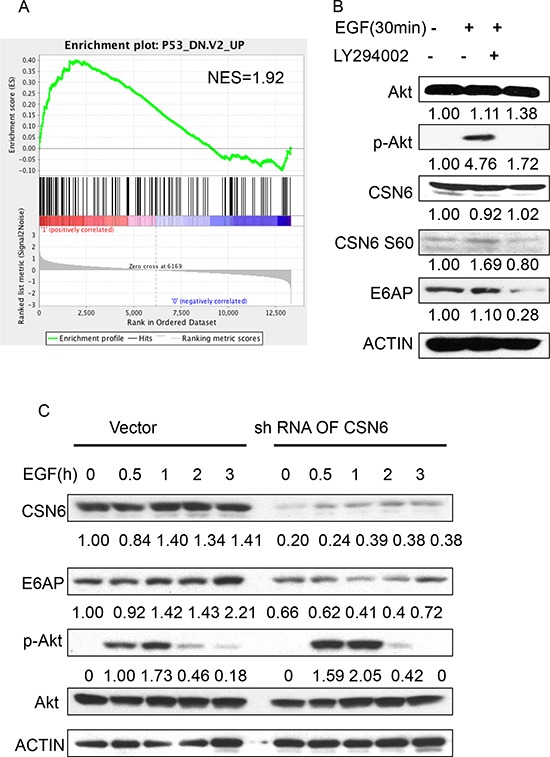
EGF-Akt signaling regulates CSN6 and E6AP stability **A.** Gene set enrichment analysis was performed in GSE6783. CSN6 overexpression correlates with genes involved in p53 down-regulated pathway. **B.** HeLa cells were treated with or without LY294002 for 1 h and then treated with EGF (100ng/nl) for 30 minutes. Equal amounts of cell lysates were immunoblotted with the indicated antibodies **C.** HeLa cells were infected with either CSN6 shRNA or control shRNA (Vector) and then treated with EGF (100 ng/ml). Equal amounts of cell lysates were immunoblotted with the indicated antibodies.

Our Gene Set Enrichment Analysis (Broad Institute) data showed a positive correlation between CSN6 overexpression and genes involved in p53 down-regulated pathway (Figure 6A), suggesting that CSN6 overexpression in cervical cancer can affect p53-mediated gene suppression. Furthermore, we found that EGF treatment increased the S60 phosphorylation level of CSN6 (Figure 6B) with concurrent Akt phosphorylation. PI3K/Akt inhibitor L294002 reduced the S60 phosphorylation level of CSN6 and caused subsequent E6AP downregulation (Figure 6B). CSN6 S60 phosphorylation is known to increase the stability of CSN6 [21]. To prove that CSN6 is critical in EGF-mediated E6AP stabilization, we performed CSN6 knockdown and analyze the steady-state expression of E6AP in the presence of EGF. CSN6 was elevated by the EGF treatment following the time course with concurrent upregulation of E6AP (Figure 6C). However, this EGF-mediated upregulation of E6AP is diminished when CSN6 is knocked down even though Akt is still activated (Figure 6C). These results suggest that E6AP is dependent on CSN6 for stabilization in the presence of EGF. Taken together, these results indicate that EGFR-PI3K-Akt signaling helps stabilize CSN6, which in turn will stabilize E6AP.

### Activation of CSN6-E6AP axis is critical for cervical cancer growth in mice

**Figure 7:**
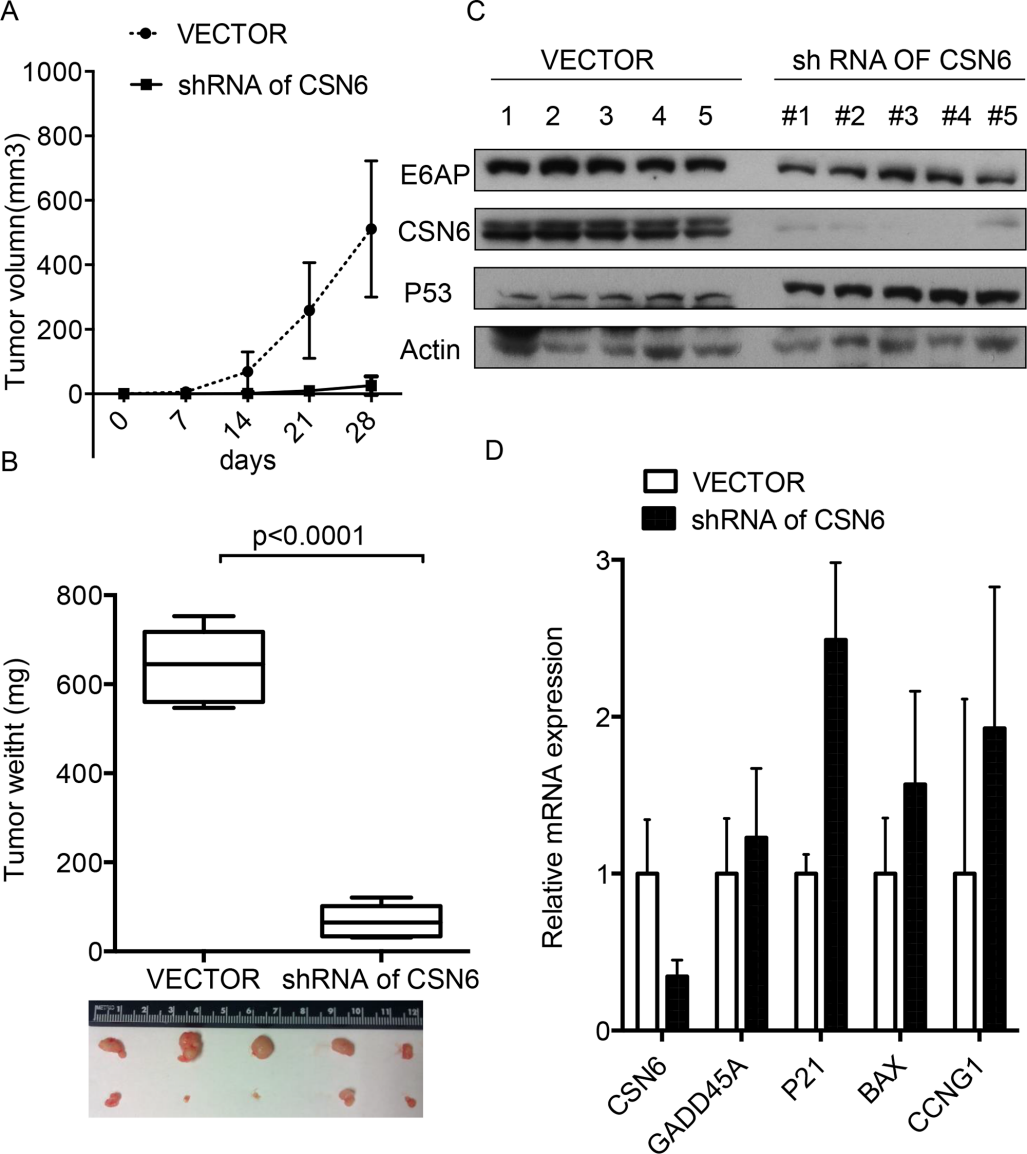
Inhibition of CSN6-E6AP axis inhibits cervical cancer growth in mice **A.** and **B.** CSN6 shRNA or control shRNA HeLa stable transfectants were subcutaneously injected into nude mice (*n* = 5). Tumor growth curve and representative tumor sizes are shown. The tumors were isolated at the end of the assay, and the tumor weight of each group was measured. Data are the means ± SDs. **C.** The tumors were isolated at the end of the assay, and tissue lysates were immunoblotted with the indicated antibodies. **D.** RNA was extracted from tumors tissues. qRT-PCR analysis was performed to measure the mRNA levels of p53 transcriptional target genes. Data are means ± SDs.

To investigate the role of the CSN6-E6AP axis in cervical cancer tumorigenesis, we injected HeLa containing CSN6 ShRNA cells into athymic nude mice. Treatment with ShRNA-CSN6 inhibited HeLa cells-mediated tumor growth (Figures 7A and 7B), reducing E6AP level and increasing p53 stabilization in tumors (Figure 7C). qRT-PCR analysis showed that p53 target genes were also upregulated after CSN6 ShRNA treatment (Figure 7D). These results strongly suggest that the CSN6–E6AP-p53 axis is regulated during the development of cervical cancer.

**Figure 8:**
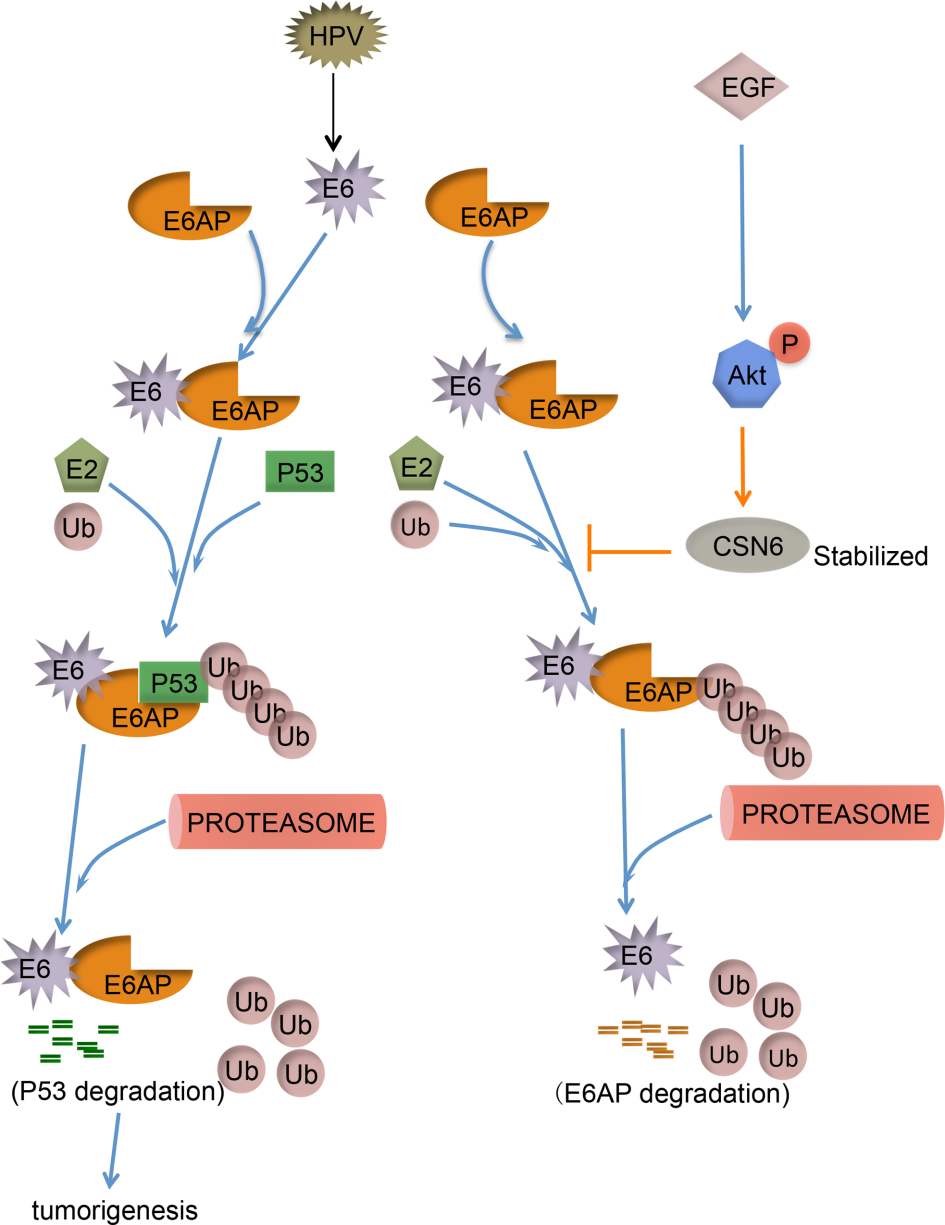
Model of CSN6 modulating E6AP stability and tumorigenesis CSN6 inhibits E6AP self-ubiquitination, which in turn stabilizes E6AP and downregulates p53, thereby facilitating tumorigenesis. EGF-Akt can stabilize CSN6 and enhance CSN6 to stabilize E6AP.

Our mechanistic studies showed that EGF/Akt-mediated CSN6 S60 phosphorylation and subse quent stabilization of E6AP thereby causing p53 downregulation and promoting cell proliferation, cell transformation as well as tumorigenesis (Figure 8). Our study fills the gap of knowledge E6AP-p53 signaling regulation in cervical cancer. Clearly, the CSN6-E6AP link serves as an important target for rational cancer therapy. Targeting the CSN6 may be a useful therapeutic strategy for cancer intervention in E6AP–deregulating cancer.

## DISCUSSION

In the present study, we found that CSN6 is elevated in cervical cancer. Both CSN6 and E3 ligase E6AP are involved in ubiquitination regulation and elevated in cervical cancer, suggesting that there is a mutual regulatory relationship involved in targeting protein degradation. In biochemical studies, high levels of CSN6 expression resulted in stabilization of E6AP in HPV positive cervical cancer cells, indicating that CSN6 has a critical role in HPV-mediated cervical cancer development. CSN6 is a critical ubiquitination regulator involved in cell cycle regulation [21, 22], but its role in cervical cancer remains unclear. We showed that CSN6 has biological activity in stabilizing E6AP, thereby diminishing p53 target gene expression; this demonstrates CSN6’s role in promoting cervical cancer development by influencing a major molecular event in this type of cancer. With this phenomenon, CSN6-E6AP-p53 axis will have potential impacts on cancer hallmarks [36] including cell proliferation [37], gemone integrity, cancer metabolism [38], cell invasion [39], or apoptosis.

Our studies showed that CSN6 functions in connection with E6AP in a circuit, resulting in the negative regulation of p53 target genes including p21 and Bax. We found that CSN6 associates with E6AP and reduces E6AP autoubiquitination, thereby stabilizing E6AP. The steady-state protein level of the E6AP protein is important in determining the ubiquitin–mediated degradation of p53, and E6AP tends to have autocatalytic degradation *in vivo*. Therefore, the effect of CSN6 in keeping the E6AP proteins stable has an impact on promoting transformation activity of HPV.

Our data show that N-terminal CSN6 is interacting with E6AP. This N-terminal CSN6 includes CSN6’s MPN domain, which is conserved in the N-terminus of yeast Mpr1 and Pad1 proteins [40–42]. Although the biological function of the MPN domain remains to be further characterized, the domain consists of polar residues that resemble the active site residues of metalloproteases [43] and is involved in a proteasome-associated activity [44]. Whether this MPN domain is critical in mitigating E6AP ubiquitination is an interesting topic to be studied. For example, it may be possible for CSN6 to antagonize other known negative regulator of E6AP, such as GRIM19 [45], which is known to enhance E6AP ubiquitination, to stabilize E6AP.

Papillomavirus E6 oncoprotein can target several cellular interacting partners for proteasome-mediated degradation. Also, E6/E6AP complex can associate with S5a, the major ubiquitin-accepting proteasome subunit [46, 47]. Here, we document the association between E6AP and CSN6. It is then possible that a complex interplay involved E6, E6AP, E6AP target proteins, and CSN6 containing COP9 signalsome will be involved in broad activities of HPV E6 to promote target protein degradation and tumorigenesis. For example, CSN6 may enhance E6-E6AP-mediated degradation of tumor suppressor protein TSC2 [12], thereby regulating the activation of mTORC1 and cap-dependent translation [48] critical for viral life cycle in epithelial tissue types and promoting neoplastic progression. Thus, our study opens a new avenue for characterizing the roles of E6/E6AP complex in papillomavirus-mediated cancer.

EGF functional polymorphism or EGF signaling is involved in influencing cervical cancer prognosis [38, 49]. EGF is also critical in promoting epithelial-mesenchymal transition (EMT) [50] in cervical cancer [51, 52]. EGFR is involved in cervical carcinoma progression [52]. Our data show that EGF/Akt signaling is causing the stabilization of E6AP through the Akt-CSN6 axis, which will contribute to carcinogenesis of cervical cancer. We previously showed that S60 of CSN6 has an Akt phosphorylation site [21] and that Akt increases the steady-state expression of CSN6 [21], which in turn will be translating into E6AP stabilization. On the basis of this, it is reasonable to assume that blocking EGF signaling will affect CSN6-E6AP axis to stop the viral life cycle and cancer transformation. E6AP-mediated p53 degradation is critical for the survival and neoplastic transformation of HPV-infected tumors; therefore, it is important to reverse this process for strategic design for the treatment of cancers with HPV infection. Effort in this area includes E6AP binding pocket inhibitors [53], and triptolide in downregulating E6 and E6AP [54]. Similarly, a strategy to antagonize CSN6-mediated E6AP stabilization will be effective. Although prophylactic HPV vaccines are available, individuals infected with HPV still need drug-based therapeutic options. Our study indicates that PI3K inhibitor in inhibiting Akt and subsequent destabilization of E6AP even under the treatment of EGF may be potential for clinical treatment of cervical cancer patients infected with HPV.

Together, our findings demonstrate that CSN6 overexpression can facilitate E6AP stabilization in cervical cancer and provide important insight into the mechanisms underlying EGF pathway deregulation in cervical cancer. That CSN6 functions as a positive regulator of E6AP prompts the idea that blocking the CSN6 signaling axis is an efficient therapeutic approach in E6AP-deregulated cervical cancer.

## MATERIALS AND METHODS

### Cell lines and reagents

HeLa and Caski (Human cervical adenocarcinoma cell line contain human papilloma virus), C33a (Human cervical epithelial carcinoma cell line with mutant p53, negative for human papilloma virus) and HEK-293T (human embryonic kidney cell line) obtained from the ATCC. Cells were maintained in Dulbecco's modified Eagle's medium/nutrient F12 media (in-house supplier) supplemented with 10% (v/v) fetal bovine serum. LY294002 was obtained from Cell Signaling (#9901). Epidermal growth factor (EGF) was obtained from Calbiochem (#324831). MG132 (C2211), cycloheximide (C4895) and Puromycin (P7255) were purchased from Sigma. Ni-NTA Argrose was obtained from Invitrogen (#R901-15). Antibodies: CSN6 (ENZO life sciences), E6AP (ENZO life Sciences), p53 (sigma), Flag (M2 monoclonal antibody, Sigma), Myc (abcam), Akt (Cell Signaling Technology), pAkt (Cell Signaling Technology), Actin (Sigma). His-Ubiquitin (UW 8610) was purchased from BioMol International. Flag-CSN6, Myc-CSN6 were previously described [28]. ShCSN6 RNA, N-CSN6 (1–170 aa) and C-CSN6 (160–327aa), CSN6 (S60A) and CSN6 (S60D) mutants were constructed as previously described [20–22].

### Transfection and generation of stable transfectants

Cells were plated in 60-mm dishes at a density of 4 × 10^5^ cell/dish 18 h before transfection. Transfection was carried out using Lipofectamine 2000 (Invitrogen) according to the manufacturer's instructions. For the generation of CSN6 knockdown cells, HeLa cells were infected with lentiviral shRNA transduction particles (GIPZ COPS6 shRNA Transfection kit, Thermo) containing either control shRNA or CSN6 shRNA. After infection, the cells were selected with 2 μg/ml puromycin for 2 weeks according to the manufacturer's protocols. The antibiotic-resistant colonies were then picked, pooled, and expanded for further analysis under selective conditions.

### Immunoblotting and immunoprecipitation

Total-cell lysates were processed as previously described [28]. Cell lysates for western blot or immunoprecipitation were collected from tissue culture dishes after being rinsed twice with cold PBS. Cells were centrifuged at low speed for 10 min, and the supernatants were discarded. The pellets were lysed with 100–300 μl 1x lysis buffer [0.5 L batch: 7.5 g 1 M Tris (Fisher), 15 ml 5M NaCl (Fisher), 0.5 ml NP-40 (USB Corp.), 0.5 ml TritonX-100 (Sigma) and 1 ml 0.5 M EDTA (Fisher)] for 20 min at 4°C. Lysis buffer also contained a cocktail of protease/phosphatase inhibitors: 5 mM NaV, 1 mM NaF, 1 μM dithiothreitol, 0.1 mg/ml Pepstatin A, 1 mM phenylmethyl sulfonyl fluoride, and 1,000x Complete Cocktail Protease Inhibitor (Roche). Lysates were immunoblotted with indicated antibodies. For immunoprecipitation, cell lysates were prepared and standardized as described above, and 1 mg protein was immunoprecipitated with appropriately diluted antibody in lysis buffer overnight. The antibody was then pulled down with 50 μl of either Protein A or G beads (Santa Cruz Biotechnology) for 1 h. The beads were centrifuged at a low speed for 10 min and the supernatant was discarded. Dried beads were mixed with 2x loading dye and boiled for 5 min. Lysate samples were loaded onto gels and sodium dodecyl sulfate polyacrylamide gel electrophoresis (SDS-PAGE) was performed.

### Ubiquitination assay

Ubiquitination was performed as previously described [55–58]. Basically, HeLa cells cotransfected with indicated plasmids were used for the experiments. At 24 h post-transfection, cells were treated with 50 μg/ml of MG132 for 6 h. The ubiquitinated proteins were pulled down with Ni-NTA Agarose. The protein complexes were then resolved by SDS-polyacrylamide gel and probed with anti-E6-AP to visualize the level of ubiquitination.

### Protein turnover assay

The cells were transfected with the indicated plasmids and incubated at 37°C with 5% (vol/vol) CO2 for 24 h. Then cycloheximide was added into the media to a final concentration of 100 μg/ml. The cells were harvested at the indicated times after CHX treatment. The protein levels were analyzed by immunoblotting.

### Cell proliferation assay

A 3-(4,5-Dimethylthiazol-2-yl)-2,5-diphenyltetrazolium bromide (MTT) assay was used to assess the cell growth rate as previously described [27]. Cells (3 × 10^3^ per well) were plated in 96-well culture plates. MTT (20 μl) was added to the cells, and the cells were incubated for 2 h. Dimethyl sulfoxide (100 μl) was then added to the cells. After incubation, the absorbance was measured at 570 nm.

### Migration assay

For the migration assay, 24-well Boyden chambers (Corning, NY) were used. Filters (8-μm pore size) precoated with fibronectin (Roche) were used for estimating cell migration. Cells were placed into the upper chamber in 0.5 ml of serum-free DMEM (1 × 105 cells per filter). DMEM supplemented with 10% fetal bovine serum was placed in the lower chamber as a chemoattractant. Migration was scored at 24 hours. Cells were fixed in methanol for 5 min at room temperature, stained with crystal violet for 5 min, and counted under microscopy. The mean numbers of cells per microscopic field over five fields per filter were calculated for triplicate experiments. Experiments were repeated at least three times.

### Quantitative polymerase chain reaction

TRizol (Invitrogen) was used to extract total RNAs from cells or tissues. The iScript cDNA Synthesis Kit (BioRad, #170-8891) was used to produce cDNA from 1 μg of RNA. Quantitative reverse-transcription polymerase chain reaction (qRT-PCR) was performed using a 7500 Real-Time PCR System (Applied Biosystems), and SYBR Green PCR Master Mix (BioRad) as previously described [59].

Primers for qRT-PCR of CSN6 (5′-TCATCGAGAG CCCCCTCTTT; 5′-CCAATGCGTTCCGCTTCCT) and p53 target genes, p21(5′-TGTCCGTCAGAACCCAT GC; 5′-AAAGTCGAAGTTCCATCGCTC), GADD45A (5′-GAGAGCAGAAGACCGAAAGGA; 5′-CACAAC ACCACGTTATCGGG), BAX (5′-CCCGAGAGGTCT TTTTCCGAG; 5′-CCAGCCCATGATGGTTCTGAT), CCNG1 (5′-GAGTCTGCACACGATAAT GGC; 5′-GTGC TTGGGCTGTACCTTCA). were as referred by Primer Banker (http://pga/mgh.harvard.edu/primerbank/). And ACTIN was used for normalization.

### FACS analysis for cell cycle and apoptosis assay

Cell cycle was analyzed following cell staining with propidium iodide (PI). Apoptosis was determined using propidium iodide (PI) and FITC-conjugated anti-Annexin V (BD Pharmingen). Cells were stained with PI and FITC-conjugated anti-Annexin V and analyzed with a FACScalibur flow cytometer.

### Xenograft mouse model

4–5 weeks old nude mice were maintained in the animal facility at the University of Texas MD Anderson Cancer Center. Mice were randomly divided into experimental groups, five for each. Different stable transfectants of HeLa cells (1 × 10^6^) in 0.1 ml PBS were injected subcutaneously into the flanks of mice. Tumor volumes were measured and recorded three times a week from day 5 of the cell inoculation. At the end of the experiment, the mice were sacrificed and the tumors were removed and weighted.

### Gene set enrichment analysis

Cancer patient data sets GSE6783, among others were obtained from the Oncomine, The Cancer Genome Atlas (TCGA) and Gene Expression Omnibus databases. These cohorts were analyzed by Gene Set Enrichment Analysis (Broad Institute) as described [2, 38, 60].
